# Diverse Humoral Immune Responses in Younger and Older Adult COVID-19 Patients

**DOI:** 10.1128/mBio.01229-21

**Published:** 2021-06-29

**Authors:** Jennifer M. Sasson, Joseph J. Campo, Rebecca M. Carpenter, Mary K. Young, Arlo Z. Randall, Krista Trappl-Kimmons, Amit Oberai, Christopher Hung, Joshua Edgar, Andy A. Teng, Jozelyn V. Pablo, Xiaowu Liang, Angela Yee, William A. Petri, David Camerini

**Affiliations:** a Department of Medicine, University of Virginia Health System, Charlottesville, Virginia, USA; b Antigen Discovery, Incorporated, Irvine, California, USA; c Department of Microbiology, Immunology and Cancer Biology, University of Virginia Health System, Charlottesville, Virginia, USA; d Department of Pathology, University of Virginia Health System, Charlottesville, Virginia, USA; e Center for Virus Research, University of California, Irvine, Irvine, California, USA; University of California, Los Angeles

**Keywords:** SARS-CoV-2, clinical severity, endemic coronaviruses, epitopes, humoral immunity, viral variants

## Abstract

We sought to discover links between antibody responses to severe acute respiratory syndrome coronavirus 2 (SARS-CoV-2) and patient clinical variables, cytokine profiles, and antibodies to endemic coronaviruses. Serum samples from 30 patients of younger (26 to 39 years) and older (69 to 83 years) age groups and with varying clinical severities ranging from outpatient to mechanically ventilated were collected and used to probe a novel multi-coronavirus protein microarray. This microarray contained variable-length overlapping fragments of SARS-CoV-2 spike (S), envelope (E), membrane (M), nucleocapsid (N), and open reading frame (ORF) proteins created through *in vitro* transcription and translation (IVTT). The array also contained SARS-CoV, Middle East respiratory syndrome coronavirus (MERS-CoV), human coronavirus OC43 (HCoV-OC43), and HCoV-NL63 proteins. IgG antibody responses to specific epitopes within the S1 protein region spanning amino acids (aa) 500 to 650 and within the N protein region spanning aa 201 to 300 were found to be significantly higher in older patients and further significantly elevated in those older patients who were ventilated. Additionally, there was a noticeable overlap between antigenic regions and known mutation locations in selected emerging SARS-CoV-2 variants of current clinical consequence (B.1.1.7, B1.351, P.1, CAL20.C, and B.1.526). Moreover, the older age group displayed more consistent correlations of antibody reactivity with systemic cytokine and chemokine responses than the younger adult group. A subset of patients, however, had little or no response to SARS-CoV-2 antigens and disproportionately severe clinical outcomes. Further characterization of these slow-low-responding individuals with cytokine analysis revealed significantly higher interleukin-10 (IL-10), IL-15, and interferon gamma-induced protein 10 (IP-10) levels and lower epidermal growth factor (EGF) and soluble CD40 ligand (sCD40L) levels than those of seroreactive patients in the cohort.

## INTRODUCTION

Coronavirus disease 2019 (COVID-19) leads to a wide range of clinical responses, varying from minor symptoms, an effective immune response, and viral clearance to major respiratory compromise, a significantly uncoordinated immune response, and death (1). Defining antibody responses, both qualitatively and quantitatively, is necessary for characterizing illness severity, assessing treatment strategies, and understanding long-term protection after vaccination.

The nucleocapsid (N) protein, a 488-amino-acid (aa) severe acute respiratory syndrome coronavirus 2 (SARS-CoV-2) internal structure that functions in compaction and protection of the viral RNA genome, and the spike (S) protein, a 1,273-aa protein that functions in the fusion of viral to host cell membranes by binding to cellular receptors, have been implicated as dominant antibody targets in COVID-19 (2–4). Correlations of antibody levels to severity of disease in previous studies have yielded mixed results owing to the heterogeneity of immune responses seen in SARS-CoV-2 infection (2, 5). There are limited data, however, on antibodies to specific epitopes within these viral proteins and their association with disease severity.

The OPEN Safely study of over 17 million patients identified common patient characteristics and comorbidities as predictors of death from COVID-19 (6). Among these, age was found to be the strongest predictor of poor outcomes. Age has also paradoxically been associated with increased antibody responses (6, 7). Other studies propose that older age promotes uncoordinated interactions between the branches of the adaptive immune response, which ultimately leads to poor outcomes (8). This suggests that the wide range of clinical presentations of COVID-19 could be attributed to multiple interactions between the components of the adaptive response, which are influenced by patient demographics and comorbidities.

Given the consistent circulation of endemic coronaviruses in the population, also known as “common cold” coronaviruses, there is interest in the cross-reactivity of antibodies directed to these viruses with SARS-CoV-2 and their subsequent effect on clinical outcomes of COVID-19 (9). The endemic human coronaviruses (HCoVs) include the alpha (HCoV-229E and HCoV-NL63) and beta (HCoV-OC43 and HCoV-HKU1) subgroups, with the latter also made up of B (containing SARS-CoV and SARS-CoV-2) and C (containing Middle East respiratory syndrome coronavirus [MERS-CoV]) lineages (9). The notable sequence homology between these subgroups raises the possibility of antibody cross-protection or enhancement with acute SARS-CoV-2 infection. More studies are needed to determine the immune interaction between responses to endemic coronaviruses and how they affect disease severity from COVID-19.

We used a novel multi-coronavirus protein microarray to identify antibody responses to small epitopes using various-sized S, envelope (E), membrane (M), N, and open reading frame (ORF) protein fragments of SARS-CoV-2. Serum samples from COVID-19 patients with mild to severe disease were exposed to these arrays, with subsequent correlation of relevant clinical data collected from medical records. Antigenic regions identified on the array were also compared to known mutations in emerging SARS-CoV-2 variants. Through the use of this multi-coronavirus protein microarray, we were additionally able to correlate the SARS-CoV-2 antibody response to those against other coronavirus subtypes and lineages.

## RESULTS

The multi-coronavirus protein microarray used in this study included four structural proteins and five accessory proteins of SARS-CoV-2 created through *in vitro* transcription and translation (IVTT): S, E, M, N, and ORFs 3a, 6, 7a, 8, and 10. Fragments of these nine proteins were made through IVTT in 50% overlapping segments of 30 aa, 50 aa, and 100 aa in length. There were additional structural proteins from SARS-CoV, MERS-CoV, HCoV-NL63, and HCoV-OC43, plus overlapping 13- to 20-aa peptides of the SARS-CoV structural proteins and the S proteins of MERS-CoV, HCoV-NL63, and HCoV-OC43 (see Table S1 in the supplemental material). Antigenic regions identified were compared to the locations of mutations of current emerging SARS-CoV-2 variants of concern (B.1.1.7, B1.351, P.1, CAL20.C, and B.1.526) (10). Serum samples were collected from COVID-19 patients of various ages and disease severities from April 2020 until July 2020. Disease severity ranged from outpatients to patients in the intensive care unit (ICU) requiring mechanical ventilation. Collection occurred during hospitalization, with emphasis on the beginning of admission if possible. The number of days from the patients’ first day of symptoms until the date of sample collection was used as an indicator of the stage of infection (Table 1). Samples were selected to represent younger adult (26 to 39 years) versus older adult (69 to 83 years) age groups and ventilated versus nonventilated patient populations. Thirty of these serum samples were used to probe the multi-coronavirus protein microarray. Clinical data, including patient medical history, clinical course, and laboratory assays, were collected from electronic medical records (Table 1; Table S2). Serum samples were additionally analyzed by Milliplex SARS-CoV-2 antigen panel 1 IgG, IgA, and IgM for comparison.

**TABLE 1:** Clinical characteristics of older and younger COVID-19 patients, with the older age group stratified by ventilation status

Parameter^*a*^	Value for group		
	Young adult patients (*n* = 10; aged 27–39 yrs)	Older patients, not ventilated (*n* = 11; aged 69–82 yrs)	Older patients, ventilated (*n* = 9; aged 69–83 yrs)
Mean age (yrs) (SD)	34.3 (3.86)	75.2 (4.79)	72.7 (4.5)

% of patients of sex			
Male	30	63.6	77.8
Female	70	36.4	22.2

Median no. of days from symptom onset^*b*^ (IQR)	8.5 (4.0)	13.0 (8.0)	14.5 (11.0)

% of patients of race	60 other	27.3 Caucasian	66.7 Caucasian
	20 Caucasian	63.6 African American	11.1 African American
	20 unknown	9.09 other	22.2 other

% of patients of ethnicity			
Hispanic	80	9.1	11.1
Non-Hispanic	10	90.9	77.8
Unknown	10		11.1

Median BMI (IQR)	27.7 (13.4)	30.0 (11.0)	27.8 (6.9)

% of patients with oxygen requirement	50 room air	45.5 room air	100 ventilated
	40 nasal cannula	54.5 nasal cannula	
	10 high flow		

Mean max temp (°C) (SD)	37.3 (0.567)	37.6 (1.01)	38.3 (0.834)

% of patients with remdesivir use^*c*^	10	9.1	11.1

% of patients with steroid use^*c*^	0	0	0

% of patients with convalescent-phase plasma use	0	0	0

% of patients with tocilizumab use	0	0	0

% of patients with clinical course	10.0 outpatient	9.1 outpatient	100 hospitalized/ICU
	90.0 hospitalized	90.9 hospitalized	
	20 ICU	27.3 ICU	

% of deceased patients	0	0	22.2

Mean length of hospital stay (days) (SD)	5.0 (3.56)	16.0 (10.7)	35.1 (25.8)

Mean comorbidity score^*d*^ (SD)	1.67 (1.29)	2.35 (1.66)	1.64 (1.53)

a IQR, interquartile range.

b Days from symptom onset, defined as the number of days from the patients’ first day of symptoms (obtained via chart review) until the date of sample collection.

c Indicates the use of a therapeutic prior to sample collection. Some patients subsequently received steroids (*n* = 4) and remdesivir (*n* = 3) after the sample was collected.

d Comorbidity score defined by the hazard ratio for death from COVID-19 infection as determined previously by Williamson et al. (6).

10.1128/mBio.01229-21.8 TABLE S1 Features of the 1st-generation ADI multi-coronavirus protein microarray. IVTT, coupled *in vitro* transcription and translation. *, RBD is the receptor binding domain (aa 319 to 541) of the SARS-CoV-2 S protein. #, the SARS-CoV-2 S protein is a stabilized form with a trimerization sequence and transmembrane domain deletion. **, the SARS-CoV S protein is a transmembrane domain-deleted form. Download Table S1, DOCX file, 0.01 MB. Copyright © 2021 Sasson et al. 2021 Sasson et al. https://creativecommons.org/licenses/by/4.0/ This content is distributed under the terms of the Creative Commons Attribution 4.0 International license.

10.1128/mBio.01229-21.9 TABLE S2 Medians and interquartile ranges of maximum laboratory values for admission of young adult, older nonventilated, and older ventilated COVID-19 patients. Download Table S2, DOCX file, 0.01 MB. Copyright © 2021 Sasson et al. 2021 Sasson et al. https://creativecommons.org/licenses/by/4.0/ This content is distributed under the terms of the Creative Commons Attribution 4.0 International license.

### Differences in antigenic reactivity to SARS-CoV-2 protein fragments in older ventilated, older nonventilated, and younger adult patients and relationship to emerging variants.

**FIG 1:**
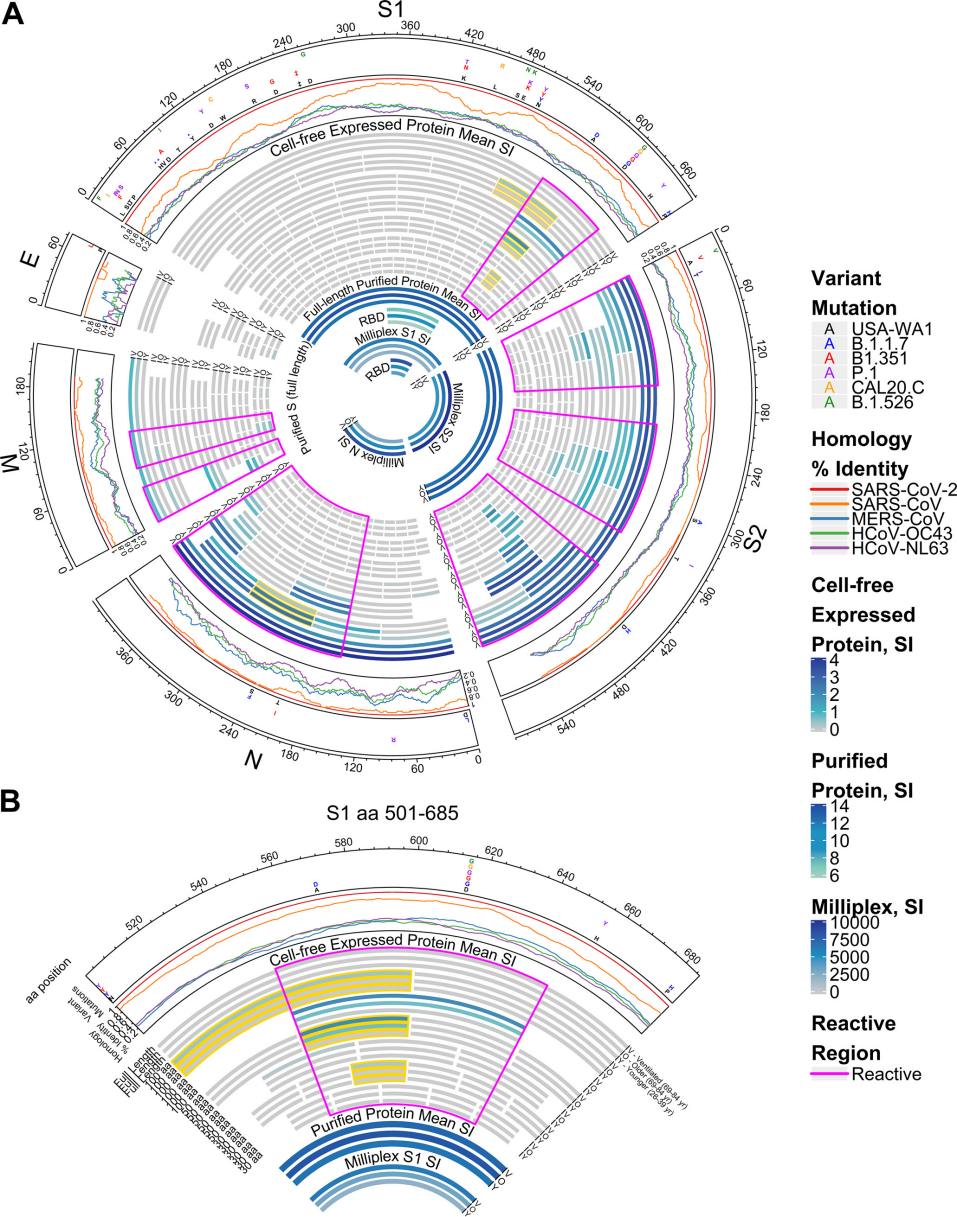
Reactivity of COVID-19 patient IgG to SARS-CoV-2 proteins displayed separately for younger, older ventilated, and older nonventilated groups. (A) Circular graphic mapping the amino acid (aa) positions of SARS-CoV-2 fragments, showing a heat map of antibody levels in each group for overlapping regions of different amino acid lengths. Proteins are indicated outside the circle plot above an axis that shows amino acid positions from the N terminus to the C terminus of each protein. The following graph moving inward shows the positions of amino acid mutations in currently circulating variants compared with the USA-WA1 variant that is represented on the protein array. ‡ at S1 aa 242 represents a 3-aa deletion from positions 242 to 244 and an R246I mutation in the B1.351 variant. Asterisks represent deletions. The following line graph shows the sequence homology of other HCoVs with SARS-CoV-2 for each gene. The inner circular heat map shows proteins and protein fragments produced *in vitro* with bars that represent the length and position of each fragment in each protein. Each fragment is drawn three times and shows the group mean normalized signal intensity (SI) of antibody binding to each fragment for COVID-19 patient serum samples in the older ventilated group (“V”) (69 to 83 years of age), the older nonventilated group (“O”) (69 to 83 years), and the younger age group (“Y”) (26 to 39 years). The IgG signal intensity is shown by a color gradient (gray to deep blue). Bar triads shown with a gold outline represent significantly differential antibody binding among all three groups, defined as a mean log_2_ signal intensity of ≥0.1 in at least one group and an unadjusted ANOVA *P* value of ≤0.05 (adjusted *P* values are provided in Tables S1 and S2 in the supplemental material). The regions of greatest reactivity for each protein are outlined in magenta. The innermost circle bands represent the responses to full-length purified recombinant S protein (shown crossing both the S1 and S2 regions) and receptor binding domain (RBD) proteins from the array. This is followed by full-length S1, S2, and N and RBD responses acquired in the Milliplex assay. (B) A sector of the circular graphic enlarged and labeled in more detail as a guide to interpreting the full figure. IgG reactivity with the C-terminal region of the S1 protein spanning aa 501 to 685 is shown.

The IgG response within the S1 protein was most notable in the region spanning aa 500 to 650 (Fig. 1A and B). The antibody reactivity to fragments in this region was significantly greater in older adult patients than in younger adult patients. Reactivity was further increased in older patients who required mechanical ventilation compared to older patients who did not require ventilation. Specifically, differences in antigenicity occurred for S1 overlapping fragments at aa 501 to 600 and aa 551 to 600, with a narrowed region of interest at aa 571 to 600. Notably, these regions include aa 570 and aa 614, which are the locations of known missense mutations in key emerging SARS-CoV-2 variants (Fig. 1A). This region also overlaps the N501Y mutation within the receptor binding domain (RBD) seen in the B.1.1.7, B1.351, and P.1 variants. Antigenic reactivity also differed significantly among the three groups within the N region spanning aa 201 to 300, which contains the S235F and T205I mutations found in the SARS-CoV-2 B.1.1.7 and B.1.351 variants, respectively. Within the S2 protein, overlapping IVTT fragments revealed three antigenic regions of interest within all three patient groups, although these were without significant differences in reactivity among the groups: S2 aa 51 to 100, S2 aa 276 to 325, and S2 aa 451 to 480. Antigenic areas in S2 also contained the mutations S982A and D1118H, which are found in the B.1.1.7 variant.

### Correlations of clinical data to antigenic regions of SARS-CoV-2 show an association of antibodies with duration of illness.

**FIG 2:**
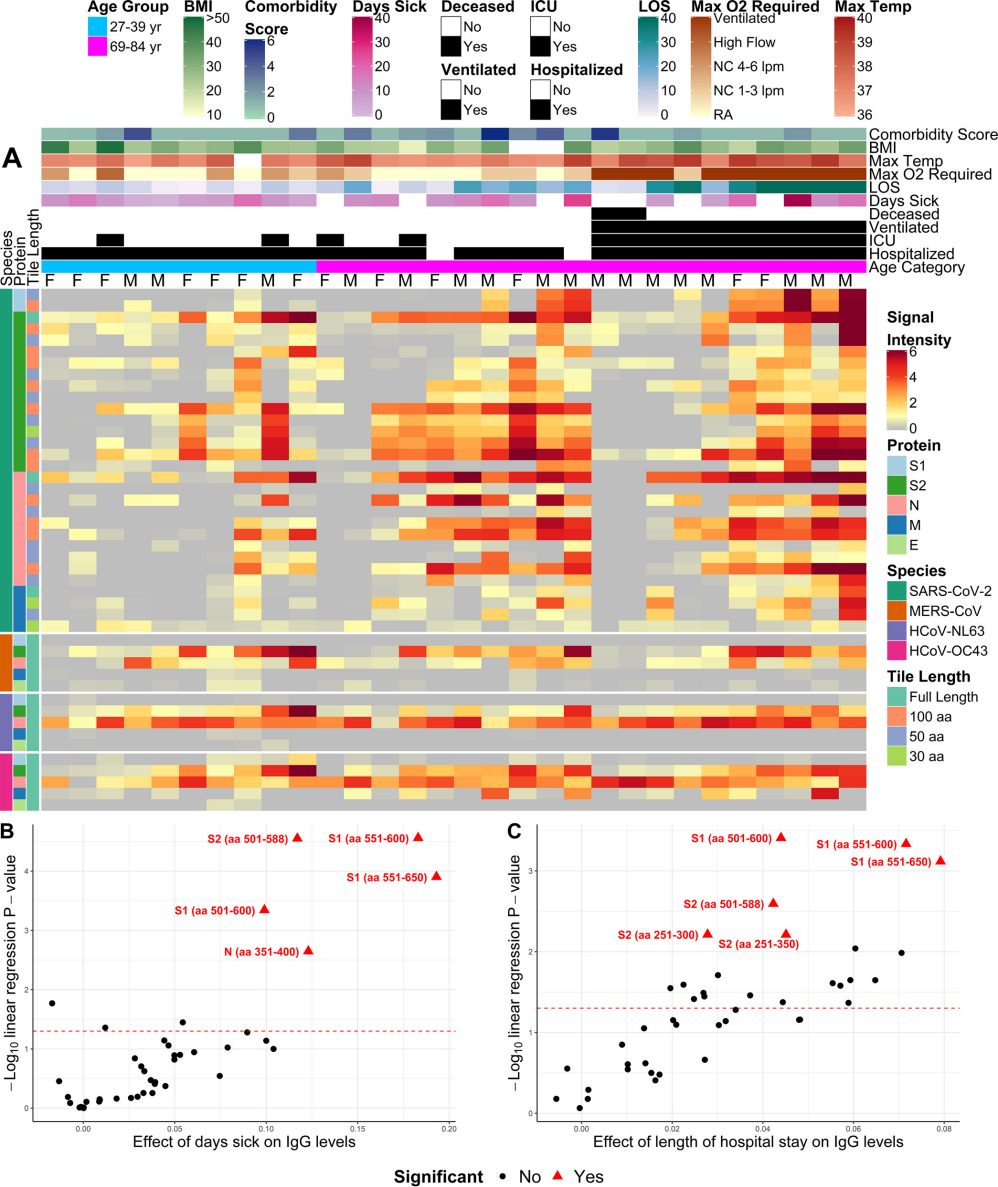
Heat map depicting relative IgG antibody responses to SARS-CoV-2 compared to other HCoVs and clinical data. (A) Heat maps presenting the signals of antibody binding to individual proteins and protein fragments within the antigenic regions of SARS-CoV-2 as well as the full-length structural proteins of MERS-CoV, HCoV-NL63, and HCoV-OC43 for individual samples. Columns represent serum samples, and rows represent proteins or protein fragments: 128 SARS-CoV-2 proteins or fragments and 5 proteins each of MERS-CoV, HCoV-OC43, and HCoV-NL63. The antibody signal intensity is shown on a color scale from gray to red. Sample clinical information is overlaid above the heat maps and includes sex (male [M]/female [F]), age category, clinical status (hospitalized, admitted to the ICU, ventilated, and/or deceased), longevity of symptoms (number of days sick prior to sample collection and length of stay [LOS] at the hospital), maximum oxygen levels required (liters per minute [LPM]), and patient measurements, including maximum body temperature, body mass index (BMI), and composite score encompassing the patient’s other comorbidities (“Comorbidity Score”). Protein/fragment information is annotated to the left of the heat maps and includes the virus, the full-length protein name, and the amino acid length of the protein fragments (“Tile Length”) (full length, or 100, 50, or 30 aa). Only fragments that were reactive (normalized log_2_ signal intensity of >1.0) in at least 10% of the study population were included in the heat map. NC, nasal cannula; RA, room air. (B and C) Volcano plots showing the statistical effect estimates of days sick prior to serum sample collection (B) and length of hospital stay (C) on IgG levels. The *x* axis shows the linear regression coefficients that were adjusted by age category, sex, and requirement of a ventilator, and the *y* axis shows the inverse log_10_ *P* values for each of the SARS-CoV-2 proteins that were reactive (normalized log_2_ signal intensity of >1.0) in at least 10% of the study population. The proteins/fragments with significant associations with length of stay and days sick after correction for the false discovery rate are highlighted as red triangles and red labels.

Patient serum reactive antibody responses to SARS-CoV-2 fragments were then arranged on a heat map for comparison to patient clinical characteristics and responses to other HCoVs (Fig. 2A). This demonstrated overall higher antibody reactivity among the older patients than among the younger patients. Moreover, antibody reactivity displayed substantial heterogeneity within age and severity groups, with some patients showing little to no IgG, IgA, and IgM response to all SARS-CoV-2 fragments (Fig. 2A; Fig. S2). Notably, patients with little to no antibody reactivity to SARS-CoV-2 did have robust reactivity to HCoV-OC43 and HCoV-NL63 proteins. Low antibody reactivity in these individuals was further validated by Millipore antibody assay levels, which confirmed that these patients had S2 and N protein reactivity in the bottom 20% of the cohort.

10.1128/mBio.01229-21.1 FIG S1 Reactivity of COVID-19 patient IgA and IgM to SARS-CoV-2 proteins displayed separately for the younger, older ventilated, and older nonventilated groups. Circular graphic mapping the amino acid (aa) positions of SARS-CoV-2 fragments, showing a heat map of antibody levels in each group for overlapping regions of different amino acid lengths. Proteins are indicated outside the circle plot above an axis that shows amino acid positions from the N terminus to the C terminus of each protein. The following graph moving inward shows the positions of amino acid mutations in currently circulating variants compared with the USA-WA1 variant that is represented on the protein array. ‡ at S1 aa 242 represents a 3-aa deletion from positions 242 to 244 and an R246I mutation in the B1.351 variant. Asterisks represent deletions. The following line graph shows the sequence homology of other HCoVs with SARS-CoV-2 for each gene. The inner circular heat map shows proteins and protein fragments produced *in vitro* with bars that represent the length and position of each fragment in each protein. Each fragment is drawn three times and shows the group mean normalized signal intensity (SI) of antibody binding to each fragment for COVID-19 patient serum samples in the older ventilated group (“V”) (69 to 83 years of age), the older nonventilated group (“O”) (69 to 83 years), and the younger age group (“Y”) (26 to 39 years). IgA and IgM signal intensities are shown by a color gradient (gray to deep blue). Bar triads shown with a gold outline represent significantly differential antibody binding among all three groups, defined as a mean log_2_ signal intensity of ≥0.1 in at least one group and an unadjusted ANOVA *P* value of ≤0.05 (adjusted *P* values are provided in Tables S1 and S2 in the supplemental material). The regions of greatest reactivity for each protein are outlined in magenta. The innermost circle bands represent the responses to full-length purified recombinant S protein (shown crossing both the S1 and S2 regions) and receptor binding domain (RBD) proteins from the array. This is followed by full-length S1, S2, and N and RBD responses acquired in the Milliplex assay. Download FIG S1, TIF file, 1.6 MB. Copyright © 2021 Sasson et al. 2021 Sasson et al. https://creativecommons.org/licenses/by/4.0/ This content is distributed under the terms of the Creative Commons Attribution 4.0 International license.

10.1128/mBio.01229-21.2 FIG S2 Heat map depicting relative IgA and IgM antibody responses to SARS-CoV-2 compared to other HCoVs and clinical data. The heat maps present the signals of antibody binding to individual proteins and protein fragments within the antigenic regions of SARS-CoV-2 as well as the full-length structural proteins of MERS-CoV, HCoV-NL63, and HCoV-OC43 for individual samples. Columns represent serum samples, and rows represent proteins or protein fragments: 128 SARS-CoV-2 proteins or fragments and 5 proteins each of MERS-CoV, HCoV-OC43, and HCoV-NL63. Antibody signal intensity is shown on a color scale from gray to red. Sample clinical information is overlaid above the heat maps and includes sex (male [M]/female [F]), age category, clinical status (hospitalized, admitted to the ICU, ventilated, and/or deceased), longevity of symptoms (number of days sick prior to sample collection and length of stay [LOS] at the hospital), maximum oxygen levels required (nasal cannula [NC], liters per minute [LPM], room air [RA]), and patient measurements, including maximum body temperature, body mass index (BMI), and composite score encompassing the patient’s other comorbidities (“Comorbidity Score”). Protein/fragment information is annotated to the left of the heat maps and includes the virus, the full-length protein name, and the amino acid length of the protein fragments (“Tile Length”) (full length or 100, 50, or 30 aa). Only fragments that were reactive (normalized log_2_ signal intensity of >1.0) in at least 10% of the study population were included in the heat map. Download FIG S2, TIF file, 0.4 MB. Copyright © 2021 Sasson et al. 2021 Sasson et al. https://creativecommons.org/licenses/by/4.0/ This content is distributed under the terms of the Creative Commons Attribution 4.0 International license.

Linear models were created to observe correlations between patient clinical data and antibody binding to SARS-CoV-2 fragments that were reactive in at least 10% of the population. After adjustment for age, sex, and ventilator status, a significant correlation was found between IgG response and days from symptom onset (DFSO) (Fig. 2B). A region of notable correlation was found in S1 aa 551 to 600, which was further supported by the significant correlation seen with S1 aa 551 to 650 and S1 aa 501 to 600. S2 aa 501 to 588 reactivity was additionally found to have a significant correlation with DFSO. There was also a significant correlation found between the IgG antibody response and length of hospital stay (Fig. 2C). As with days of illness, this correlation was found to be most notable regarding the S1 region spanning aa 551 to 600, further supported by the significant correlation of the S1 fragment from aa 551 to 650 and the S1 fragment from aa 501 to 600. An additional correlation with length of hospital stay was seen in S2 aa 501 to 588.

### Serum cytokine and chemokine profiles correlate with antibodies in older adult COVID-19 patients more than in younger adult patients.

We then assessed the association between antibody reactivity to SARS-CoV-2 fragments adjusted for age, sex, and ventilator status and cytokine levels in each patient sample analyzed with the Milliplex MAP (multi-analyte profiling) human cytokine/chemokine/growth factor panel (48-plex) (Fig. S3). There were significantly positive correlations seen in IgG, IgM, and IgA antibody responses and levels of interleukin-5 (IL-5), tumor necrosis factor beta (TNF-β), platelet-derived growth factor AB/BB (PDGF-AB/BB), epidermal growth factor (EGF), soluble CD40 ligand (sCD40L), and interleukin-17A (IL-17A) in serum samples. Negative correlations were seen between antibody responses and levels of interleukin-10 (IL-10), interferon gamma (IFN-γ)-induced protein 10 (IP-10) (or CXC chemokine ligand 10 [CXCL10]), IFN-α2, tumor necrosis factor alpha (TNF-α), and interleukin-15 (IL-15).

10.1128/mBio.01229-21.3 FIG S3 Correlation of reactive SARS-CoV-2 proteins and fragments with selected cytokine levels. The heat map shows Pearson’s correlation coefficients between antibody and cytokine levels on a colorimetric scale from negative correlations in green to positive correlations in red. The antigens displayed correspond to proteins and fragments produced *in vitro* that were seropositive (normalized signal intensity of ≥1.0) in at least 10% of the study population (i.e., at least 3 individuals). The cytokines displayed were selected based on significant associations with antibody levels in linear mixed-effects regression models, adjusted for age category, sex, and requirement of a ventilator. Significances of the correlations are shown by overlaid asterisks (*, *P* < 0.05; **, *P* < 0.005; ***, *P* < 0.0005). IL-17A and IL-5 were selected due to significant associations with individual antibody responses. Protein/fragment information is annotated to the right of the heat maps and includes the protein name, the amino acid coordinates in parentheses, and the length of the protein fragments (“Tile Length”) (full length or 100, 50, or 30 aa). Download FIG S3, TIF file, 0.7 MB. Copyright © 2021 Sasson et al. 2021 Sasson et al. https://creativecommons.org/licenses/by/4.0/ This content is distributed under the terms of the Creative Commons Attribution 4.0 International license.

**FIG 3:**
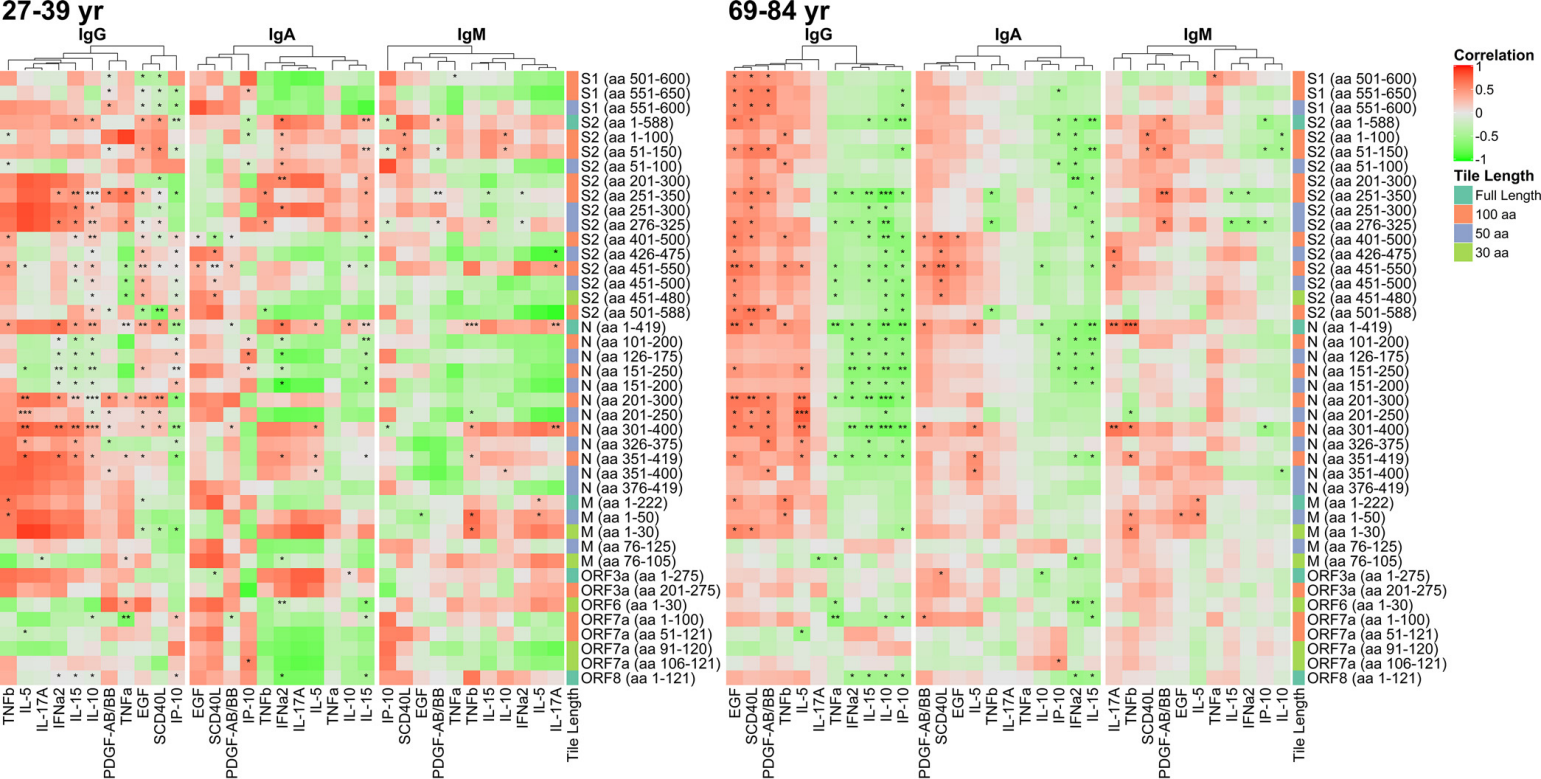
Correlation of reactive SARS-CoV-2 proteins and fragments with selected cytokine/chemokine levels stratified by age group. The heat map shows Pearson’s correlation coefficient between antibody and cytokine levels on a colorimetric scale from negative correlations in green to positive correlations in red. Significances of the correlations are shown by overlaid asterisks (*, *P* < 0.05; **, *P* < 0.005; ***, *P* < 0.0005). Plots are separated by the younger age group (26 to 39 years of age; *n* = 10) (left) and the older age group (69 to 83 years; *n* = 20) (right). The antigens displayed correspond to proteins and fragments produced *in vitro* that were seropositive (normalized log_2_ signal intensity of ≥1.0) in at least 10% of the study population. The cytokines displayed were selected based on significant associations with antibody levels in linear mixed-effects regression models, adjusted for age category, sex, and requirement of a ventilator. IL-17A and IL-5 were selected due to significant associations with individual antibody responses in ordinary least-squares regression models adjusted for age category, sex, and ventilator. Protein/fragment information is annotated to the right of the heat maps and includes the protein name, the amino acid coordinates in parentheses, and the lengths of the protein fragments (“Tile Length”).

Correlations between antibody reactivity to antigenic protein fragments and cytokine/chemokine levels in patient serum samples were then stratified by age group (Fig. 3). This revealed the same positive and negative correlations consistently represented in the older age group. In contrast, the younger adult patient group demonstrated notable heterogeneity in its correlations with antibody responses. Furthermore, correlations that were significantly positive or negative in the older age group at times showed a reverse correlation in the younger age group. For instance, IL-10 was significantly negatively correlated with the IgG response, most notably to N and S2 fragments, in the older age group. This, however, was not consistent with the younger age group, where correlations between the IgG response to these regions and IL-10 were, although variable, mostly positive. However, similar correlations did occur regardless of age group, such as with the significantly positive correlation seen between the IgG response to N aa 200 to 400 and the levels of IL-5.

### IgG responses to SARS-CoV-2 S2 proteins correlate with IgG responses to homologous endemic human coronavirus S2 proteins.

**FIG 4:**
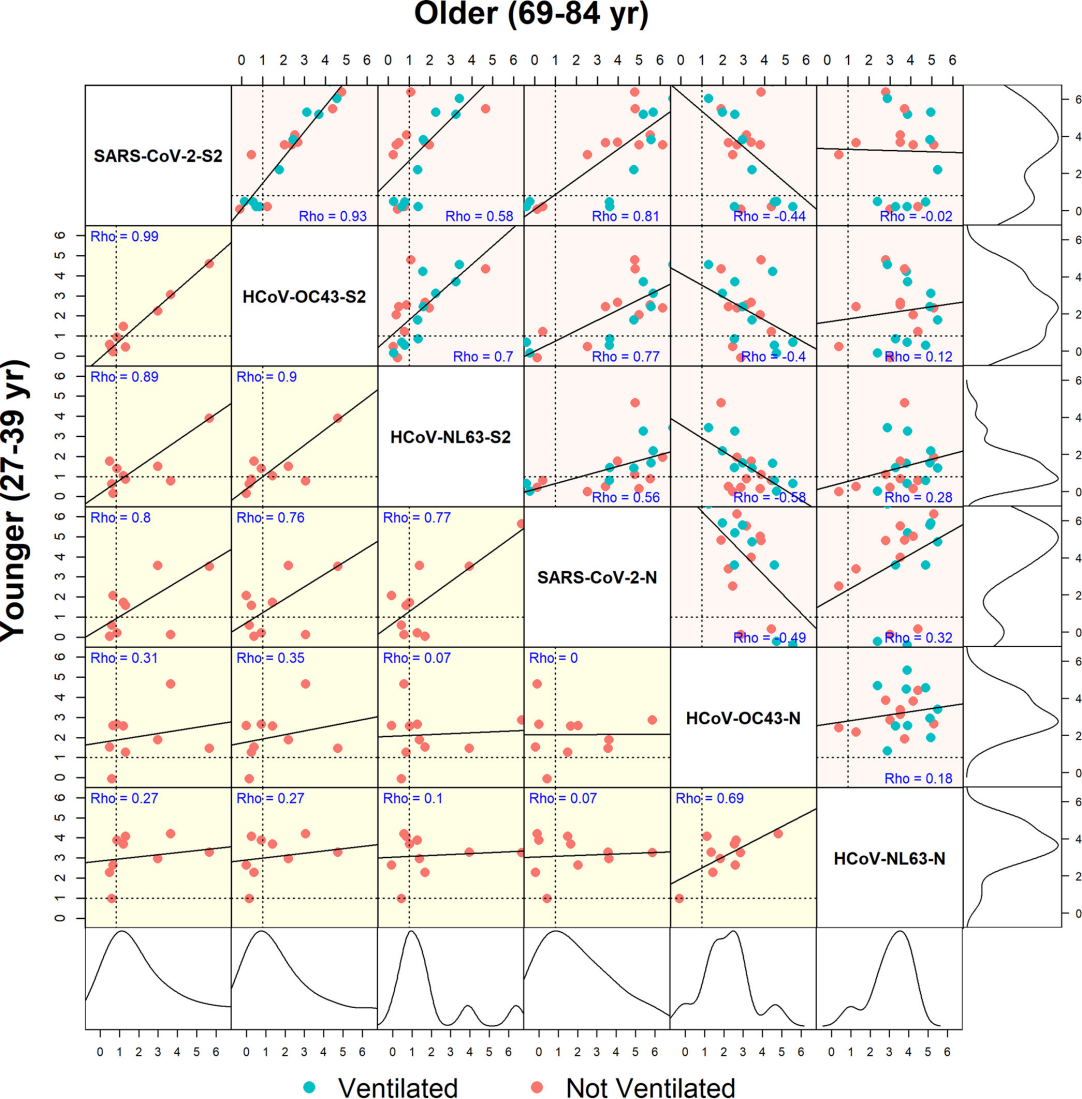
Correlation of IgG responses to full-length proteins of SARS-CoV-2 and two endemic human coronaviruses. A correlogram depicts Pearson’s rank correlation coefficient (rho) between IgG-normalized signal intensities for SARS-CoV-2, HCoV-OC43, and HCoV-NL63 full-length S2 and N proteins produced *in vitro*. The lower left half of the diagonal (shaded in yellow) shows correlations between the reactivities of sera from the younger age group (27 to 39 years of age), and the upper right half of the diagonal (shaded in pink) shows the older group (69 to 84 years). Lines of seropositivity defined as a normalized log_2_ signal intensity of ≥1 are depicted by horizontal and vertical dotted lines within each scatterplot. Slow-low responders are represented by dots that fall below these dotted lines. The rho coefficient is listed in blue lettering in each box. The outermost right and bottom boxes represent density plots for the older and younger age groups, respectively.

We further assessed the correlation of the IgG responses to the S2 and N proteins of SARS-CoV-2 to those of HCoV-OC43 and HCoV-NL63 (Fig. 4). There were strong linear correlations between antibody reactivities to the S2 protein of SARS-CoV-2 and the S2 proteins of HCoV-OC43 and HCoV-NL63 regardless of age or ventilator status (Fig. 4; Fig. S3A and B). This was inconsistent with correlations observed between IgG responses to N proteins, which were weakly correlated, in part due to individuals with little or no reactivity (normalized log_2_ signal intensity of <1.0), which suggested a population with either delayed or negative seroreactivity, referred to here as “slow-low”-responding individuals. This was further exemplified through density plots highlighting the differences in bimodal antibody responses between responding and slow-low-responding individuals. Notably, the slow-low SARS-CoV-2 responders were responsive to S2 and N proteins of endemic HCoVs and appeared to be nonresponsive solely to SARS-CoV-2 proteins (Fig. 4).

Positive correlations between S2 responses to SARS-CoV-2 and endemic HCoVs were not observed for IgM, largely owing to the limited IgM response seen to HCoV-OC43 and HCoV-NL63 as discussed above (Fig. S5A). While correlations similar to those with IgG were seen when comparing IgA responses to S2 proteins of endemic HCoVs, this did not extend to N proteins, again due to the limited IgA response in serum (Fig. S5B). When comparing the antibody responses to individual SARS-CoV-2 fragments and endemic coronaviruses, there were diffusely positive correlations between S2 fragments and S2 proteins of endemic coronaviruses, most notably to HCOV-OC43 (Fig. S6).

10.1128/mBio.01229-21.4 FIG S4 Correlation between IgG responses to SARS-CoV-2 full N and S2 proteins and other endemic human coronaviruses stratified by age and severity. Correlograms depict Pearson’s correlation coefficients (ρ) between IgG normalized signal intensities to SARS-CoV-2, HCoV-OC43, and HCoV-NL63 N and S2 full-length proteins produced *in vitro*. In panel A, the lower left half of the diagonal shows the correlation between the reactivities of sera in the younger age group (27 to 39 years of age), and the upper right half of the diagonal shows the older group (69 to 84 years). In panel B, the lower left half of the diagonal shows the correlations between the reactivities of sera in the nonventilated group, and the upper half of the diagonal shows the ventilated group. The color scale indicates positive correlations in darker shades of blue and negative correlations in darker shades of red, and ρ is overlaid on each comparison. Additionally, the narrowness and slope of the ellipses represent increasing positive or negative correlations. Download FIG S4, TIF file, 0.4 MB. Copyright © 2021 Sasson et al. 2021 Sasson et al. https://creativecommons.org/licenses/by/4.0/ This content is distributed under the terms of the Creative Commons Attribution 4.0 International license.

10.1128/mBio.01229-21.5 FIG S5 Correlation between IgA and IgM responses to SARS-CoV-2 full N and S2 proteins and other endemic human coronaviruses. Correlograms depict Pearson’s correlation coefficients (rho) between IgM (A) and IgA (B) normalized signal intensities to SARS-CoV-2, HCoV-OC43, and HCoV-NL63 N and S2 full-length proteins produced *in vitro*. The lower left half of the diagonal shows correlations between the reactivities of sera in the younger age group (27 to 39 years of age), and the upper right half of the diagonal shows the older group (69 to 84 years). Ventilated patients are represented by teal dots, and nonventilated patients are represented by red dots. The line of seropositivity, defined as a normalized log_2_ signal intensity of ≥1, is depicted by horizontal and vertical dotted lines in each scatterplot. The rho coefficient is listed in blue lettering in each box. Download FIG S5, TIF file, 0.3 MB. Copyright © 2021 Sasson et al. 2021 Sasson et al. https://creativecommons.org/licenses/by/4.0/ This content is distributed under the terms of the Creative Commons Attribution 4.0 International license.

10.1128/mBio.01229-21.6 FIG S6 Correlation of antibody responses to SARS-CoV-2 fragments and endemic coronaviruses stratified by age. The heat map depicts Pearson’s correlation coefficients between antigenic antibody fragments and endemic coronaviruses on a colorimetric scale from negative correlations in green to positive correlations in red. The antigens displayed correspond to proteins and fragments produced *in vitro* that were seropositive (normalized signal intensity of ≥1.0) in at least 10% of the study population (i.e., at least 3 individuals). Significances of the correlations are shown by overlaid asterisks (*, *P* < 0.05; **, *P* < 0.005; ***, *P* < 0.0005). Heat maps are stratified by younger (27 to 39 years of age) (left) and older (69 to 84 years) (right) age groups and by IgG, IgA, and IgM reactivities (subpanels from left to right within each age group). SARS-CoV-2 fragments are compared to full-length S2 and N from HCoV-OC43 and HCoV-NL63. Protein/fragment information is annotated to the right of the heat maps and includes the protein name, the amino acid coordinates in parentheses, and the length of the protein fragments (“Tile Length”) (full length or 100, 50, or 30 aa). Download FIG S6, TIF file, 0.4 MB. Copyright © 2021 Sasson et al. 2021 Sasson et al. https://creativecommons.org/licenses/by/4.0/ This content is distributed under the terms of the Creative Commons Attribution 4.0 International license.

**FIG 5:**
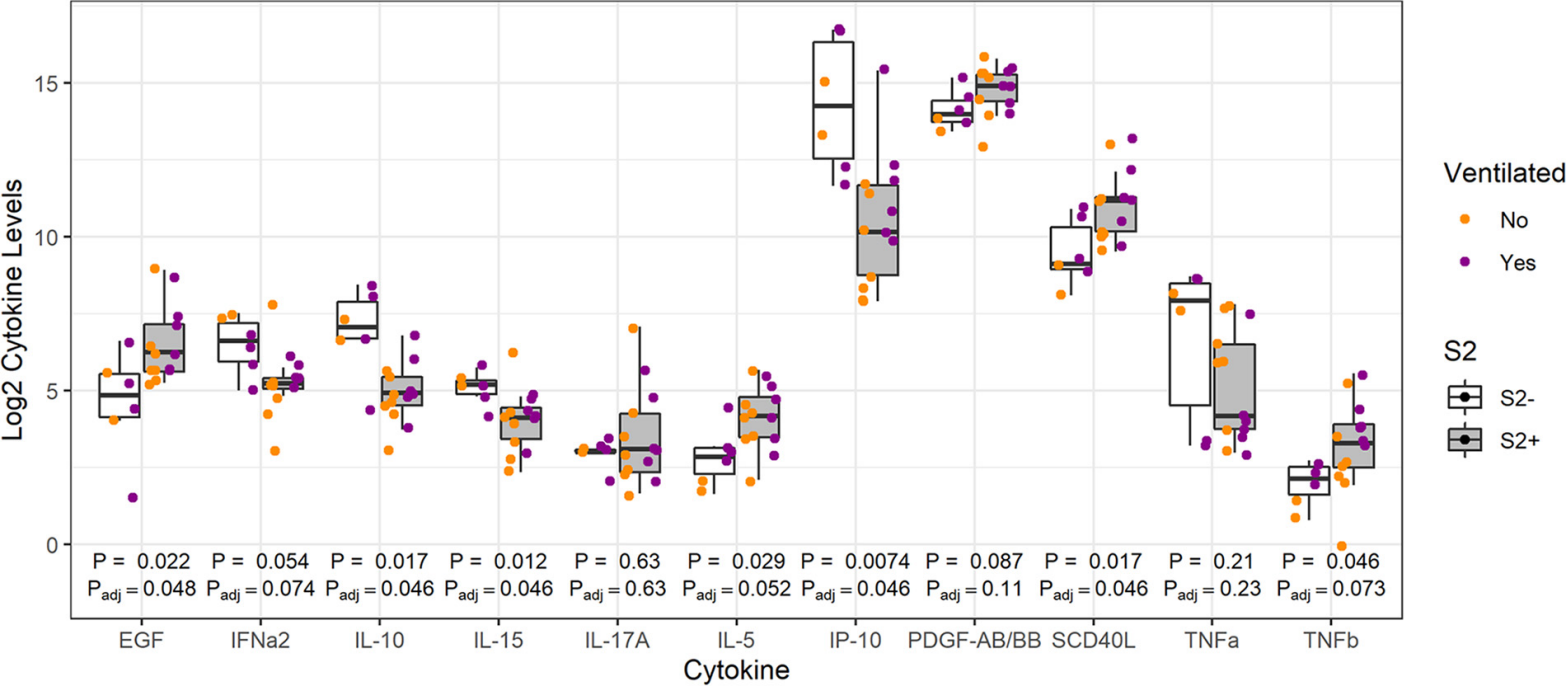
Differences in cytokine and chemokine levels between slow-low responders and seroreactive older adult COVID-19 patients. The box plot illustrates the top 11 differences in cytokine levels of those not responding to SARS-CoV-2 full-length S2 proteins (S2^−^) (“slow-low responders”) and those responding (S2^+^) (“seroreactive”). Cytokine/chemokine levels are plotted on a log_2_ scale. Unadjusted Wilcoxon’s rank sum *P* values are denoted below each pair of boxes, with an asterisk above *P* values that remain significant after correction for the false discovery rate.

Given the notable separation seen in slow-low-responding individuals compared to the rest of the cohort, cytokine profiles were then assessed in these patients. As the majority of these individuals were in the older age group, cytokines/chemokines from the older age group were compared (Fig. 5). We identified four slow-low-responding individuals to all SARS-CoV-2 proteins (N^−^ and S2^−^), with an additional two patients who were seronegative to S2 (S2^−^) but seroreactive to N (N^+^) (Fig. S7). Analysis of cytokine/chemokine responses in all six patients revealed significantly higher IL-10, IL-15, and IP-10 levels in slow-low responders than in the rest of the older age group. It also displayed significantly lower levels of EGF and sCD40L in slow-low responders (Fig. 5).

10.1128/mBio.01229-21.7 FIG S7 Antibody responses to SARS-CoV-2 fragments in older adult patients separated by seroreactive and slow-low-reactive patients to the full-length S2 fragment. The heat map presents the signals of antibody binding to individual protein fragments produced *in vitro* within the antigenic regions of SARS-CoV-2 separated by patients who are seroreactive to the full S2 protein produced *in vitro* (S2^+^) and those who are seronegative to the full-length S2 protein produced *in vitro* (S2^−^). Columns represent serum samples within the older adult age group. The antibody signal intensity is shown on a color scale from gray to red. Fragments displayed correspond to proteins and fragments produced *in vitro* that were seropositive (normalized log_2_ signal intensity of ≥1.0) in at least 10% of the study population. Download FIG S7, TIF file, 0.2 MB. Copyright © 2021 Sasson et al. 2021 Sasson et al. https://creativecommons.org/licenses/by/4.0/ This content is distributed under the terms of the Creative Commons Attribution 4.0 International license.

## DISCUSSION

This study showed greater antibody reactivity and associated consistent cytokine/chemokine profiles to specific SARS-CoV-2 epitopes in older patients with more severe disease than in a younger cohort. The uniformity of epitopes recognized by patients within our cohort and their overlap mutations identified in emerging viral variants may have implications particularly when considering the possibility of SARS-CoV-2 reinfection. We additionally demonstrated a considerable correlation in anti-S2 reactivities regardless of age and severity between SARS-CoV-2 and other endemic HCoVs, which questions the role that preexisting HCoV antibodies may have in acute COVID-19 infection. Finally, we characterized a subset of patients with little to no antibody response, allowing for discussion of the clinical consequences and potential therapeutic options for patients with this immunological phenotype.

Antigenic regions identified in our study were similar to those found by Camerini et al. in a companion study that looked at the differences in antibody responses to the array between COVID-19-negative and -positive patients (11). Among the antibody-reactive regions identified in our study was S1 aa 551 to 600, an area just past the RBD, near the C terminus of the S1 protein, and three regions within the S2 protein, aa 51 to 100, aa 276 to 325, and aa 451 to 480, all of which have been implicated in previous studies involving SARS-CoV-2 epitope mapping (12, 13). In our study, IgG reactivity to the S1 region spanning aa 551 to 600 not only was highest in the older, ventilated COVID-19 patient cohort but also was significantly correlated with hospital length of stay and days of illness. Differences found in antigenicity to this region between older, ventilated patients and older, nonventilated patients likely result from a longer duration of disease in the ventilated patients. Previous studies have also shown a correlation between IgG responses to S1 protein and days of illness, which likely can be attributed, at least in part, to reactivity within this region (14).

Epitopes unveiled in this study are relevant in the discussion of some of the key rising SARS-CoV-2 variants and raise the possibility that their antigenicity provides evolutionary pressure for a variant competitive advantage. Although numerous variants have been identified at this point, those of currently suggested clinical consequence (i.e., B.1.1.7, B1.351, P.1, CAL20.C, and B.1.526) have defining mutations that overlap the antigenic regions discussed in this study. Evidence concerning the reinfection capability of these viral variants in addition to the limited efficacy of current vaccines and antibody cocktail therapies has begun to emerge (15). Although we did not complete neutralization assays to reveal the quality of antibody responses in these patient samples, future studies will compare the serological responses to the original strain epitopes to those of new variants to provide insights into the potential for reinfection by SARS-CoV-2 mutants.

When antibody reactivity to SARS-CoV-2 was correlated with cytokine/chemokine profiles in these serum samples, we found that consistent correlations existed within the older patient group, while large variations occurred in younger COVID-19 patients. This suggests a clinically unfavorable cytokine/chemokine profile that correlates with higher antibody reactivity, which more commonly occurs in older patients. IL-5, a type 2 (Th_2_) cytokine shown by Lucas et al. to correlate with severe COVID-19 disease, had a significantly positive correlation to the antibody response to N aa 200 to 400, which may further suggest poor outcomes related to Th_2_ responses (16). Additionally, IL-10 had significant negative correlations to antibody responses to S2 and N proteins in the older age group, which is consistent with its known anti-inflammatory properties. Interestingly, IL-10 has been implicated in numerous other viral, bacterial, and protozoal infections whose clinical outcomes were observed to be time dependent on peak IL-10 production and its ability to cause either inhibition of effective pathogen clearance or prevention of excessive immune responses to microbial antigens (17).

We also found strong correlations between the IgG responses to SARS-CoV-2, HCoV-NL63, and HCoV-OC43 S2 proteins, which have also been noted in previous studies (12, 13). This has been attributed to considerable sequence homology between the S2 proteins of SARS-CoV-2 and endemic HCoVs, particularly to the more closely related endemic betacoronaviruses (HCoV-OC43 and HCoV-HKU1). Associations found in this study suggest either cross-reactivity of newly produced antibodies to SARS-CoV-2 with other HCoV antigens in the array or cross-reactivity in which preexisting antibodies to other coronaviruses can recognize SARS-CoV-2 antigens. While this is difficult to determine without analysis of patient sera prior to infection, we see evidence of both phenomena occurring in our cohort. The lack of a concomitant serum IgM response observed in this cohort to endemic HCoVs along with the lack of a correlation between the IgM responses to S2 proteins between them suggest preexisting, boosted IgG rather than new, acute antibody production. However, the magnitude of reactivity to S2 protein and the positive correlation of anti-S2 IgG between SARS-CoV-2 and endemic HCoVs suggest a component of new antibody reactivity to some epitopes due to significant immune activation. Preexisting, cross-reacting antibodies to SARS-CoV-2 would allow an opportunity for cross-neutralization of SARS-CoV-2 antigens and raise the possibility of improved clinical outcomes in these patients. However, of note, in our analysis, correlations of antibodies to S2 proteins between SARS-CoV-2 and endemic HCoVs were apparent regardless of age or ventilator status, suggesting less of an influence on clinical outcomes.

There was also notably absent antibody reactivity to SARS-CoV-2 proteins among a subset of individuals in this cohort, none of whom were immunosuppressed prior to COVID-19 diagnosis. Given that these patient samples were collected at a single time point, it is difficult to know if these represent patients with no response throughout the illness course or are individuals in whom antibody levels were slow to respond. Wajnberg et al. found the latter in an assessment of longitudinal samples, noting that in addition to a slow antibody response to SARS-CoV-2 infection, peak titers were lower than those in patients with a more robust initial response (18). This may be clinically relevant as patients with low and slow antibody responses may be those who are likely to benefit the most from SARS-CoV-2 antibody treatment regimens.

We further characterized slow-low responders by looking at their clinical characteristics and immune responses. Although a conclusive analysis is limited by sample size, two out of the four slow-low responder (N^−^ and S2^−^) older adults required ventilation, three were admitted to the ICU, and two were the only deceased patients in the study, suggesting negative clinical outcomes associated with a minimal antibody response in these patients. The small sample size was further compounded by missing data, particularly in gathering the number of days since symptom onset, which was likely due in part to the difficult nature of clinical history gathering in patients with critical illness. As this is a key component in differentiating between a rising, early-disease antibody level and a truly delayed antibody response, it is difficult to confidently conclude delayed seroreactivity in these individuals without additional longitudinal samples. Nevertheless, one out of the four individuals had these timing data and was determined to be 11 days from symptom onset to sample collection, which suggests a delayed antibody response in this patient.

In contrast to severe COVID-19 disease linked with high antibody responses as discussed above, slow-low responders suggest an alternative immunological profile to infection providing an additional avenue for poor clinical outcomes. Among the cytokine differences discovered between slow-low responders and responsive patients, IL-10 was implicated as one of the most differential, with slow-low responders displaying significantly higher levels than the rest of the older patient cohort. This is again congruent with the known influences of IL-10 and highlights its potential role in the slow-low responder patient phenotype (17). We additionally found a significant decrease in sCD40L in slow-low responders compared to the rest of the group, consistent with sCD40L’s ability to promote B cell proliferation and differentiation and immunoglobulin production (19).

A substantial limitation of our study was the small sample size, which limited our ability to detect relationships between epitopes, cytokines, and clinical outcomes. This further limited our ability to statistically analyze and classify slow-low responder samples, which were therefore categorized subjectively based on reactivity with full-length N and S2 proteins. The small sample size also restricted our ability to analyze the age variable in multiple strata and/or on a continuum, which future studies with larger sample sizes should address. Furthermore, differences in clinical metadata, including race/ethnicity and gender, that existed between the older and younger age groups limited our ability to make conclusions without confounding influence. While age has been widely recognized as the most significant predictor of disease severity in COVID-19, many additional characteristics are known to impact outcomes in COVID-19 and likely also the immune response to infection. Furthermore, the discrepancy in DFSO between the age groups may have had an impact on humoral responses. As with many small-cohort studies, there was notable variation in the clinical metadata, especially in the DFSO, with a large influence from one or two outliers. The top outliers in DFSO fell into our older age group, where sample collection began farther into clinical course due to either transfer from an outside facility and requirement of extensive ventilation or admission due to delayed, prolonged recovery. Patients with outlier values for DFSO did not appear to cluster by sex, race/ethnicity, or clinical course variables.

Our study was also limited by the use of proteins and fragments produced in Escherichia coli in addition to proteins made in eukaryotic cells. This restricts our ability to see epitopes that require eukaryotic posttranslational modifications such as glycosylation in antigens made in E. coli. This is particularly relevant with regard to the S1 protein, which is highly glycosylated (20–22). The lack of reactivity with patient sera may occur because these regions are covered by glycans in the native structure and/or because the lack of glycosyl groups does not allow the proteins and fragments produced *in vitro* to assume their native conformations (20). Nevertheless, we did see immunoreactivity with regions of the S1 and S2 proteins that contain N-linked glycans (21, 22). Moreover, we were able to detect an area in S1, aa 551 to 600, that is notable for its higher antigenicity in older, ventilated patients and its correlations with days of illness and length of stay, as discussed above. Finally, our study did not evaluate for proinflammatory IgG Fc structures of antibodies produced to array proteins, which have been linked to COVID-19 disease severity (23). Overall, despite these limitations, this study was able to provide significant insight into the complex immunological process that accompanies SARS-CoV-2 infection. Future studies should investigate antibody responses of patients who recovered from wild-type SARS-CoV-2 infection to responses to SARS-CoV-2 variant proteins in a next-generation array, which may reveal different antigenic regions than those presented in this study. Additionally, further studies should explore slow-low responders on a larger scale with attention to clinical outcomes and characterization of associated immune pathways to further address the significance of this phenotype and the potential need for correction with antibody therapies.

## MATERIALS AND METHODS

### Patient samples and clinical data collection.

Patients who tested positive for COVID-19 by PCR at the University of Virginia Medical Center had residual routine laboratory specimens collected into a biorepository. Serum samples of patients were collected from April 2020 until July 2020. Blood collected in EDTA was centrifuged at 1,300 × *g* for 10 min, and plasma was then aliquoted and stored at −80°C. Thirty of these serum samples were provided to Antigen Discovery, Inc. (ADI).

Clinical data, including patient medical history, laboratory work, and clinical course, were collected from the electronic medical record using an honest broker with unique study numbers to ensure confidentiality (Table 1; see also Table S2 in the supplemental material). This honest broker served to independently collect clinical metadata for the 30 patients in this cohort and allow the remainder of the research team to interpret the data in a deidentified fashion. The number of days from symptom onset was determined with assistance from the honest broker who read through history and physical examination notes, emergency department notes, progress notes, and discharge summaries for patients with COVID-19. Comorbidity scores were derived from hazard ratios presented previously by Williamson et al. to appropriately weigh patient comorbidities with previously observed associations in the risk of death from COVID-19 (6). The collection of blood samples and deidentified patient information was approved by the University of Virginia Institutional Review Board (IRB-HSR numbers 22231 and 200110).

### Protein microarray analysis of serum samples.

The first-generation multi-coronavirus protein microarray, produced by ADI (Irvine, CA, USA), included 935 full-length coronavirus proteins; overlapping 100-, 50-, and 30-aa protein fragments; and overlapping 13- to 20-aa peptides from SARS-CoV-2 (WA-1), SARS-CoV, MERS-CoV, HCoV-NL63, and HCoV-OC43. Purified proteins and peptides were obtained from BEI Resources. SARS-CoV-2 and SARS-CoV S proteins were made in Sf9 insect cells, and the SARS-CoV-2 RBD was made in HEK-293 cells. Other proteins and protein fragments were expressed using an E. coli *in vitro* transcription and translation (IVTT) system (rapid translation system; Biotechrabbit, Berlin, Germany) and printed onto nitrocellulose-coated glass Avid slides (Grace Bio-Labs, Inc., Bend, OR, USA) using an Omni Grid Accent robotic microarray printer (Digilabs, Inc., Marlborough, MA, USA). Microarrays were probed with sera, and antibody binding was detected by incubation with fluorochrome-conjugated goat anti-human IgG, IgA, or IgM (Jackson ImmunoResearch, West Grove, PA, USA, or Bethyl Laboratories, Inc., Montgomery, TX, USA). Slides were scanned on a GenePix 4300A high-resolution microarray scanner (Molecular Devices, Sunnyvale, CA, USA), and raw spot and local background fluorescence intensities, spot annotations, and sample phenotypes were imported and merged in R (R Core Team, 2017), in which all subsequent procedures were performed. Foreground spot intensities were adjusted by subtraction of local background, and negative values were converted to a value of 1. All foreground values were transformed using the base 2 logarithm. The data set was normalized to remove systematic effects by subtracting the median signal intensity of the IVTT controls for each sample. With the normalized data, a value of 0.0 means that the intensity is no different than the background, and a value of 1.0 indicates doubling with respect to the background. For full-length purified recombinant proteins and peptide libraries, the raw signal intensity data were transformed using the base 2 logarithm for analysis.

### Milliplex serum analysis.

Data from the protein microarray were compared to data for the same 30 samples analyzed with Milliplex SARS-CoV-2 antigen panel 1 IgG, IgA, and IgM (Millipore-Sigma, St. Louis, MO). IgG, IgA, and IgM antibodies were captured by specific bead region microspheres, each conjugated with SARS-CoV-2 S1, S2, RBD, or N, and measured by the median fluorescence intensity (MFI). Kit instructions were followed. Samples were diluted 1:100 in assay buffer. Ninety-six-well plates were prewetted with 200 μl wash buffer and incubated for 10 min. Twenty-five microliters of assay buffer was added to all wells. Twenty-five microliters of each diluted sample was added to the sample wells. Sixty microliters of both sonicated (30 s) and vortexed (1 min) analytes and control beads was combined and brought to a final volume of 3 ml with the addition of assay buffer. After vortexing, 25 μl of the bead mixture was dispensed into each plate well and incubated for 2 h at room temperature. A handheld magnetic plate washer was used to retain magnetic beads, while liquid contents were discarded. Fifty microliters of phycoerythrin–anti-human immunoglobulin (IgG, IgA, or IgM per the kit in use) detection antibody was added to each well, and the mixture was incubated for 90 min at room temperature. Plates were washed with a magnetic plate washer before and after detection antibody addition. One hundred fifty microliters of sheath fluid was added to each well, and the mixture was shaken at room temperature for 5 min. The plate was then read on a Luminex Magpix instrument system with a minimum of 50 beads of each analyte collected per well. Cytokine levels were additionally measured in each sample via Milliplex MAP human cytokine/chemokine/growth factor panel A (48-plex) using similar methods.

### Statistical analysis.

Analysis of variance (ANOVA) was used for comparison of the individual antibody response means among the younger, older ventilated, and older nonventilated groups. Proteins or protein fragments expressed using the IVTT system were classified as reactive antigens based on a normalized signal intensity seropositivity threshold of 1.0 and a seroprevalence cutoff of 10% of the study population for IgG, IgA, or IgM. Multivariable ordinary least-squares (OLS) regression was used to model associations between antibody and patient information. Antibody responses to individual reactive antigens (*n* = 52) were modeled as dependent variables, and the following variables were modeled as independent variables: sex, age category, requirement of a ventilator, number of days symptomatic prior to sample collection, length of hospital stay, admission to the ICU, maximum required supplemental oxygen category, comorbidity score, maximum body temperature while admitted, body mass index (BMI), maximum C-reactive protein (CRP), maximum ferritin, maximum D dimer, minimum lymphocytes, maximum aspartate transaminase (AST) and troponin laboratory levels, and the base 2 log-transformed measurements from the Milliplex serum analysis. Due to the moderate sample size of the study, not all independent variables were modeled simultaneously. Three “base” variables were used to adjust the effect estimates of all other independent variables in separate 4-variable models; these base variables were sex, age category, and requirement of a ventilator. Adjustment for the false discovery rate was performed using the “p.adjust” function in R (24). To select variables associated with SARS-CoV-2-specific antibodies, linear mixed-effects regression (LMER) was used to model all antibody responses against SARS-CoV-2-reactive antigens with random intercepts at the sample level and antigen level to adjust for repeated measures. Similar to the approach with OLS regression, LMER models used the same 3 base variables to fit separate models for all other fixed-effects variables. All coefficients were returned from models fit using restricted maximum likelihood (REML). To generate *P* values for LMER models, the models were refit using maximum likelihood (ML) and compared by ANOVA against null models with the coefficient removed using ML. Cytokines and chemokines that were significantly associated with antibody levels in LMER models were correlated with SARS-CoV-2-reactive antigens using Pearson’s correlation coefficient. Clinical patient variables were associated with cytokine levels using OLS regression, similarly to antibody models. The correlation between SARS-CoV-2 S2 and N proteins and HCoV-OC43 and HCoV-NL63 S2 and N proteins was assessed using Pearson’s correlation coefficient. Samples were categorized as “slow-low responders” if full-length S2 IgG responses had a normalized signal intensity of less than 1.0. Differences in median log_2_ cytokine levels between slow-low responder and seroreactive subjects were assessed using Wilcoxon’s rank sum test. Data visualization was performed using the circlize (25), ComplexHeatmap (26), ggplot2, and corrplot (27) packages in R. The *P* values presented for full-length and overlapping fragments of SARS-CoV-2 proteins were not adjusted for the false discovery rate because the measurements are not independent, and an appropriate method of *P* value correction was not, to our knowledge, available for the extent of dependence in the antibody measurements. As expected, there were high levels of colinearity in the antibody responses to overlapping fragments of different sizes in the reactive regions of SARS-CoV-2 proteins. Although unadjusted *P* values were used in these comparisons, the concordance of fragment antibody binding and differential immunoreactivity in the independent study reported concurrently by Camerini et al. lends confidence that the responses reported are unlikely to be due to chance (11). However, further studies will be able to validate these findings.

### Data availability.

All the data generated and/or analyzed supporting the findings of this study are available within the article, the supplemental material, or an external public data repository. IgG, IgA, and IgM signal intensity data from the protein microarrays used in the study are available in an external data repository (28). Clinical metadata corresponding to patient serum used in the array and Milliplex antibody, chemokine, cytokine, and growth factor data are also included in the repository (28).
