# Supplementary material for: Diverse Humoral Immune Responses in Younger and Older Adult COVID-19 Patients
Source: mBio. 2021 Jun 29;12(3):e01229-21. doi: 10.1128/mBio.01229-21 (PMC8262923; doi:10.1128/mBio.01229-21)
Supplement: TABLE S1 [file mbio.01229-21-st001.docx]

**Table S1. Features of the 1^st^ Generation ADI Multi-Coronavirus Protein Microarray**

| **Virus** | **S-protein** | **E-protein** | **M-protein** | **N-protein** | **Accessory or Other Proteins** | **Total Number of Features = 1007** |
| --- | --- | --- | --- | --- | --- | --- |
| SARS-CoV-2 | RBD*, S^#^ from BEI, S1, S2, 30, 50 & 100 aa fragments by IVTT at ADI | Protein and 30, 50 aa fragments by IVTT at ADI | Protein and 30, 50, 100 aa fragments by IVTT at ADI | Protein BEI, 30, 50 & 100 aa fragments by IVTT at ADI | ORF 3a, 6, 7a, 8 and 10 protein, 30, 50 & 100 aa fragments by IVTT at ADI | 11 proteins, 311 fragments = 322 features |
| SARS-CoV | Protein** and peptide set from BEI Resources | IVTT by ADI | Protein, peptides from BEI | Protein, peptides from BEI | 3CL protease | 5 proteins, 208 peptides = 213 features |
| MERS-CoV | BEI Resources | IVTT by ADI | IVTT by ADI | BEI Resources | ORF 3a, 4a, 4b, 5 and 8b protein IVTT by ADI | 9 proteins |
| HCoV-NL63 | Protein by IVTT at ADI, peptides from BEI | IVTT by ADI | IVTT by ADI | IVTT by ADI | ORF 3 protein IVTT by ADI | 5 proteins and 226 peptides = 231 features |
| HCoV-OC43 | Protein by IVTT at ADI, peptides from BEI | IVTT by ADI | IVTT by ADI | IVTT by ADI | HE, N2 protein IVTT by ADI | 6 proteins and 226 peptides = 232 features |

IVTT means coupled *in vitro* transcription and translation. * RBD is the receptor binding domain, aa 319 to 541 of the SARS-CoV-2 S protein. ^#^ SARS-CoV-2 S protein is a stabilized form with a trimerization sequence and transmembrane domain deletion. ** SARS-CoV-S protein is a transmembrane domain deleted form.
