# Supplementary material for: Diverse Humoral Immune Responses in Younger and Older Adult COVID-19 Patients
Source: mBio. 2021 Jun 29;12(3):e01229-21. doi: 10.1128/mBio.01229-21 (PMC8262923; doi:10.1128/mBio.01229-21)
Supplement: TABLE S2 [file mbio.01229-21-st002.docx]

**Table S2. Median and interquartile range of maximum lab values for admission of the young adult, older non ventilated, and older ventilated COVID-19 patients.**

|  | **Young Adult Patients**  **(N=10; Age 27-39)** | **Older Patients, Not Ventilated**  **(N=11; Age 69-82)** | **Older Patients, Ventilated**  **(N=9; Age 69-83)** |
| --- | --- | --- | --- |
| **C-Reactive Protein (mg/dL)** | 7.70 (0.9-12.7) | 7.85 (5.15-13.3) | 9.80 (1.8-24.1) |
| **Ferritin (ng/mL)** | 188 (64-925) | 704 (407-993) | 2596 (1238-4003) |
| **D dimer (ng/mL)** | 345 (203-461) | 219 (169-580) | 1882 (1049-2356) |
| **Minimum Lymphocyte Count (K/ul)** | 1.02 (0.52-1.07) | 0.90 (0.840-1.32) | 0.620 (0.43-1.44) |
| **Aspartate**  **Aminotransferase (U/L)** | 60.0 (43.5-98.5) | 42.5 (36-65) | 74.0 (70-114) |
| **Troponin (ng/mL)** | 0.030 (-) | 0.030 (0.03-0.08) | 0.040 (0.03-0.17) |
