# Supplementary figures and images for: Diverse Humoral Immune Responses in Younger and Older Adult COVID-19 Patients
Source: mBio. 2021 Jun 29;12(3):e01229-21. doi: 10.1128/mBio.01229-21 (PMC8262923; doi:10.1128/mBio.01229-21)

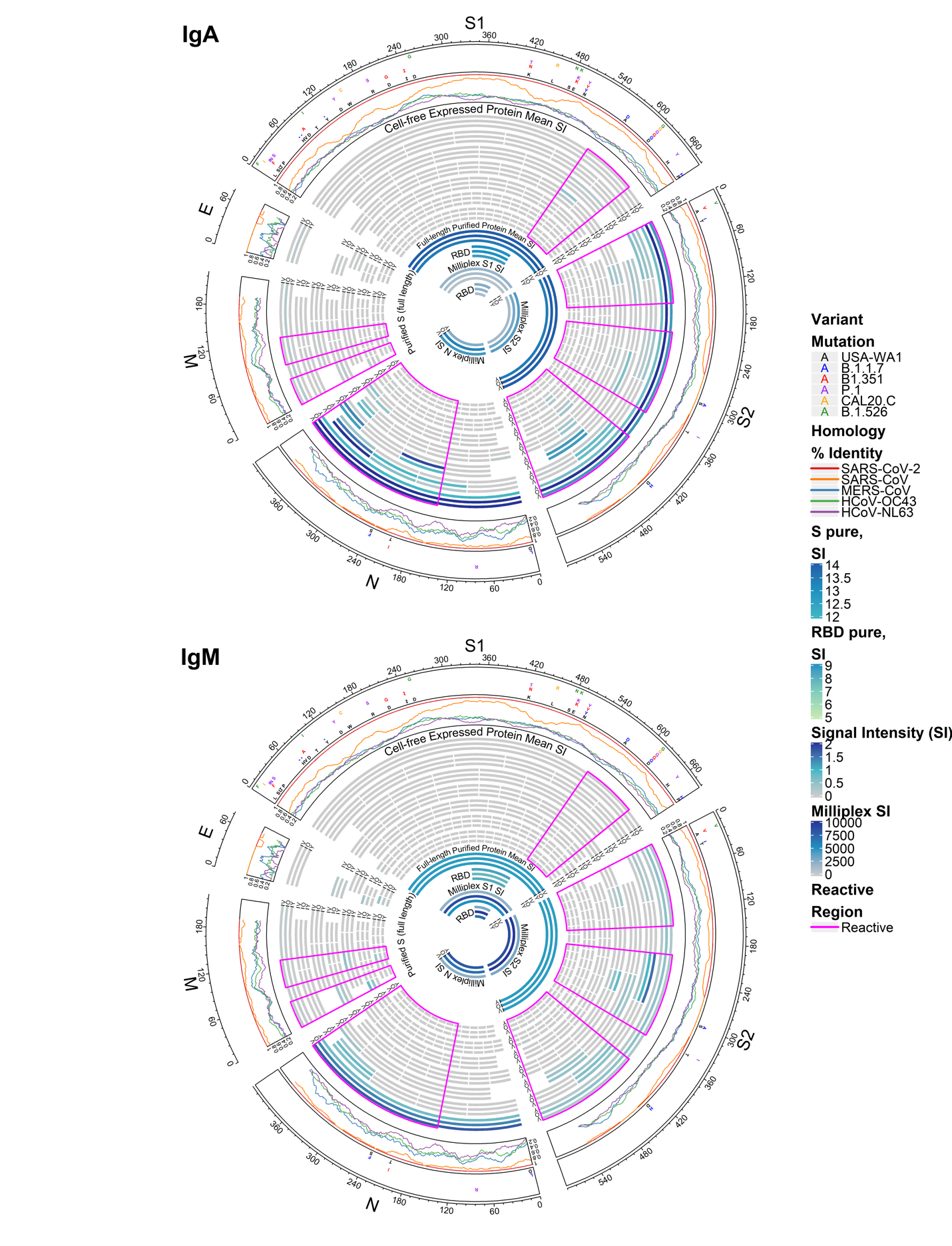

Supplement: FIG S1 [file mbio.01229-21-sf001.tif]

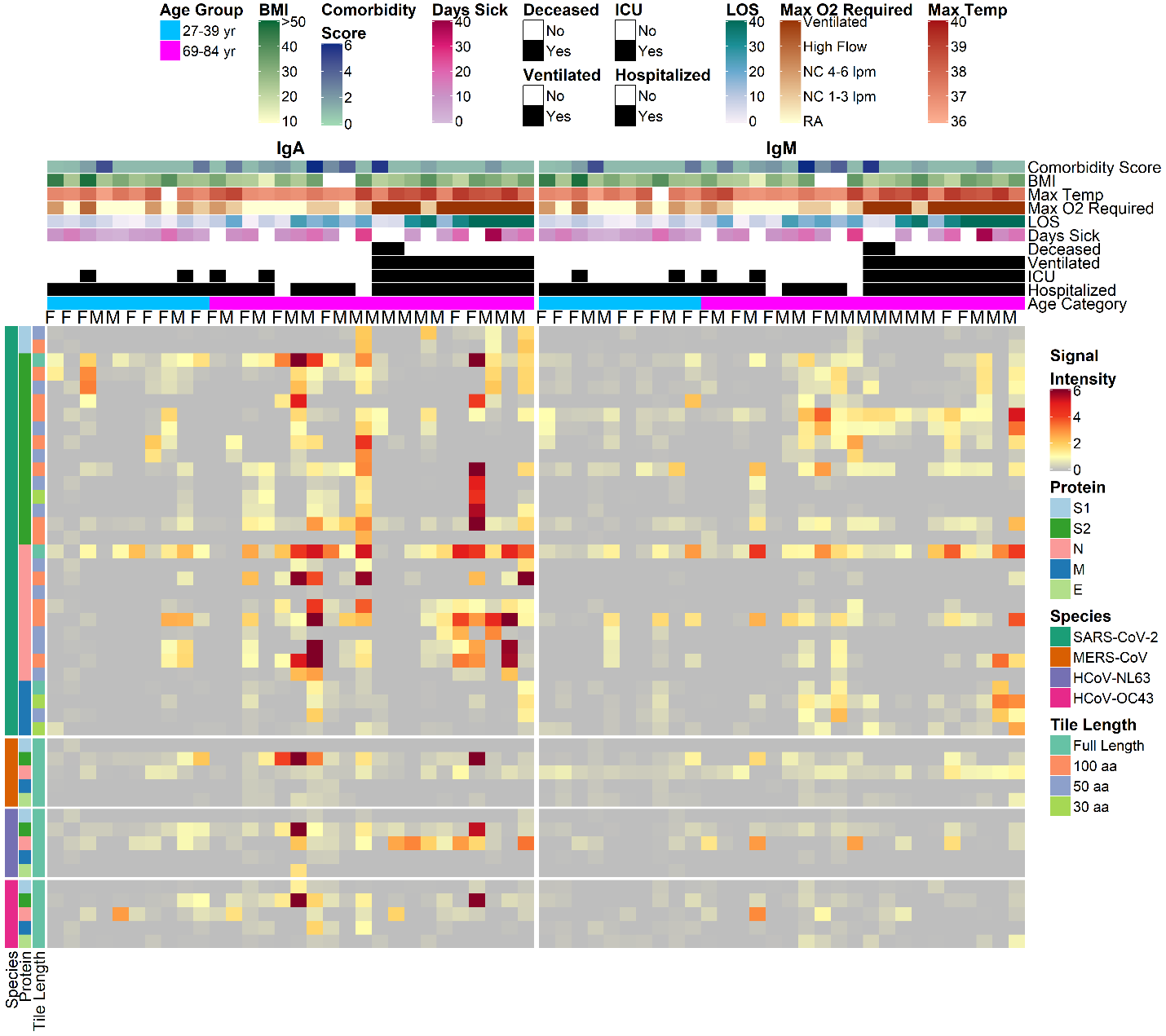

Supplement: FIG S2 [file mbio.01229-21-sf002.tif]

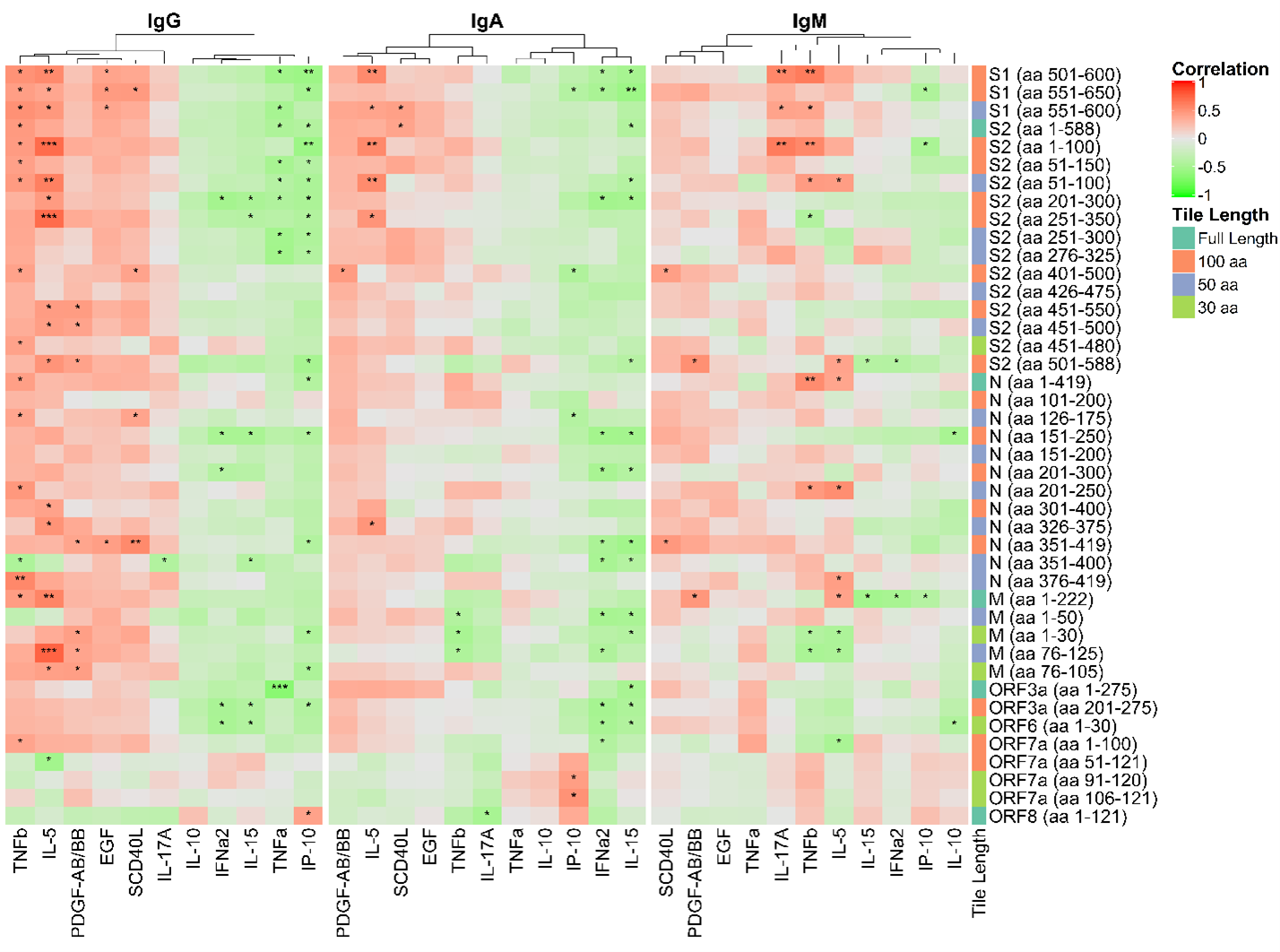

Supplement: FIG S3 [file mbio.01229-21-sf003.tif]

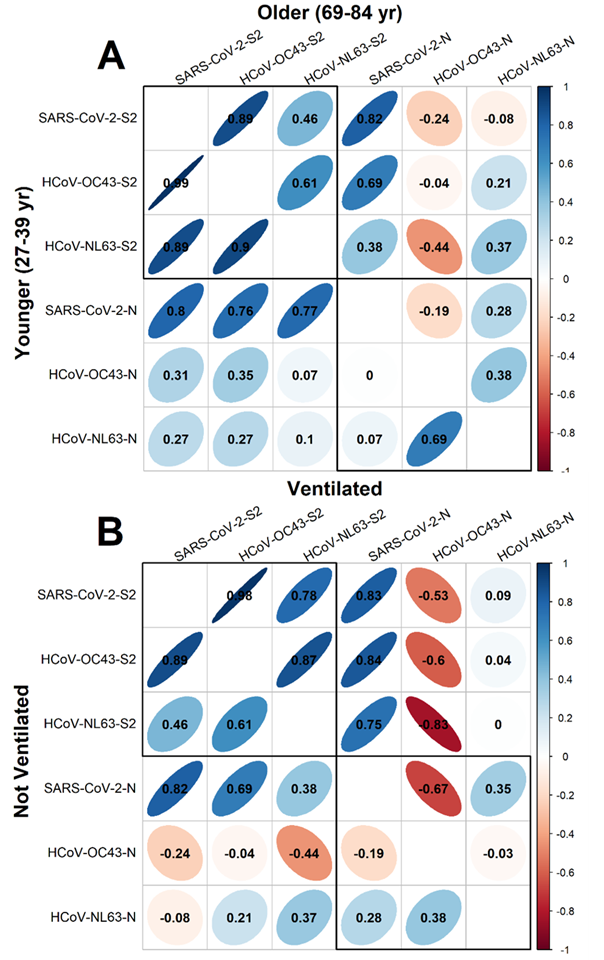

Supplement: FIG S4 [file mbio.01229-21-sf004.tif]

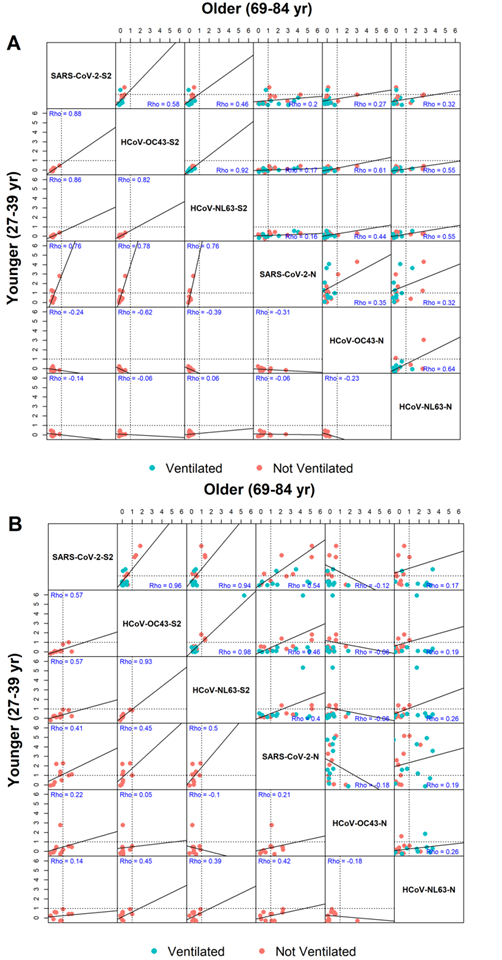

Supplement: FIG S5 [file mbio.01229-21-sf005.tif]

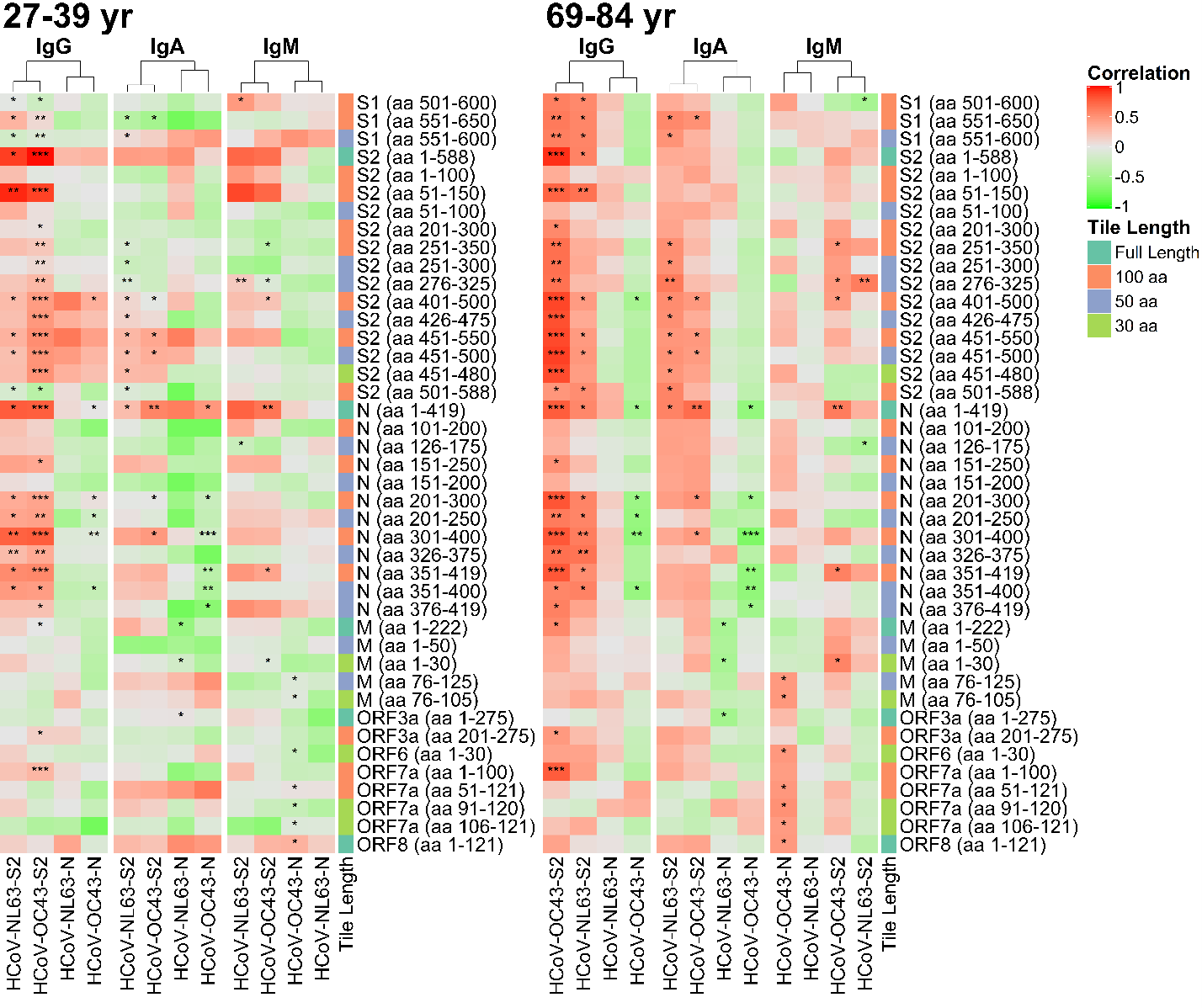

Supplement: FIG S6 [file mbio.01229-21-sf006.tif]

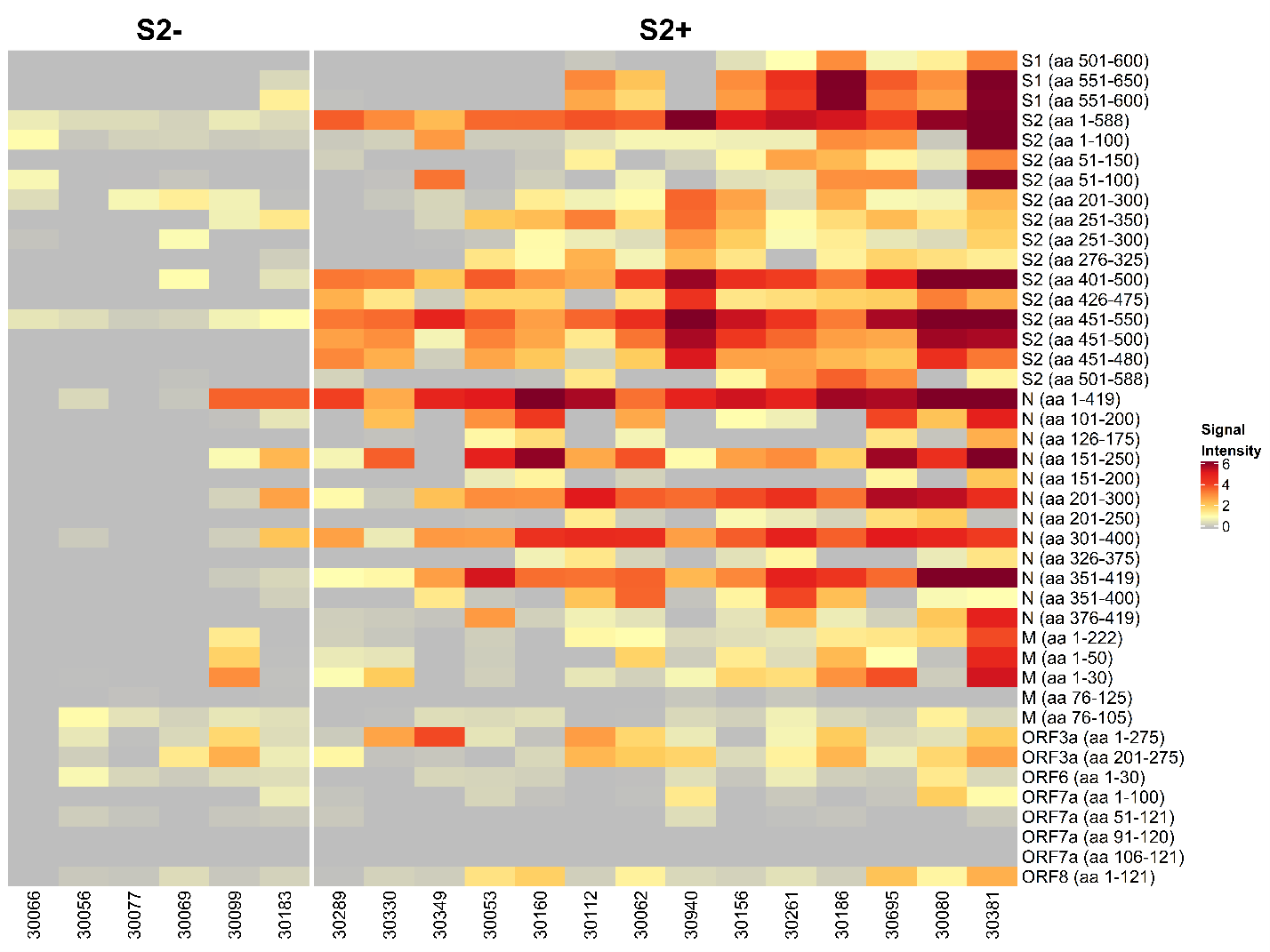

Supplement: FIG S7 [file mbio.01229-21-sf007.tif]
